# Analysis of the Underwater Radiated Noise Generated by Hull Vibrations of the Ships

**DOI:** 10.3390/s23021035

**Published:** 2023-01-16

**Authors:** Rodrigo F. Javier, Ramis Jaime, Poveda Pedro, Carbajo Jesus, Segovia Enrique

**Affiliations:** 1 Department of Physics, Systems Engineering and Signal Theory, University of Alicante, Ctra. San Vicente del Raspeig, 03080 Alicante, Spain; 2 Department of Civil Engineering, University of Alicante, Ctra. San Vicente del Raspeig, 03080 Alicante, Spain

**Keywords:** underwater acoustic pollution, shipping noise, hull vibrations, structure-borne noise, radiation efficiency, transfer function, underwater radiated noise

## Abstract

Shipping traffic is recognised as the main man-noise source of the anthropogenic noise generated in the marine environment. The underwater acoustic pollution is increased due to the increment of the human activity at seas supposing a threat for marine habitats. The ship as acoustic source must be understood and controlled to manage the maritime areas both in time and space to reduce the impact of noise in marine fauna. Shipping noise is mainly composed of flow noise, propeller noise and machinery noise. This research is focused on the analysis and estimation of the underwater radiated noise generated by the vibrations of the onboard machinery or structure-borne noise based on the calculation of the transfer function. This function relates the acceleration levels of the vibrations of the hull’s panels and the radiated noise by them using the radiation efficiency. Different analytical methods to estimate the radiation efficiency are presented and compared with data collected at sea. The measurements are performed acquiring simultaneously acceleration and acoustic levels by means on accelerometers installed on the hull’s panels at different positions and hydrophones deployed close to the bow, middle and stern of the ship. The analysis of the transmission of the vibrations along the ships is performed using the data from different locations of the hydrophones. The quality of the measurements is analysed using the coherence function through the spectral correlation between the measurement of vibrations and acoustic levels. On the other hand, signal-to-noise ratio is computed to verify the strength of the noise sources. The computed transfer function is used to predict the underwater radiated noise from vibrations showing differences less than 2 dB re to 1 μPa^2^.

## 1. Introduction

The main source of underwater acoustic pollution is shipping traffic [1,2,3,4,5] reaching average levels of 138 dB re to 1 μPa^2^ [6]. The rise in underwater noise levels caused by increasing shipping traffic in terms of both number of ships and gross tonnage [7] may have negative effects, both physical and behavioural, on marine organisms. 

The International Maritime Organization (IMO) has recognised the negative impact of excessive underwater noise levels [8], introducing voluntary guidelines aimed at ship designers, shipyards, and operators, with the goal of reducing the contribution of commercial shipping to underwater acoustic pollution. Control of the Underwater Radiated Noise (URN) generated by ships is a critical issue in order to reduce the underwater noise pollution generated by shipping traffic.

Shipping noise is a combination of tonal sounds and broadband noise spread over the frequency range between 2 Hz and 100 kHz [3,9]. A ship is a complex noise source composed of the superimposition of several contributions. The main sources of the radiated noise by a ship are the induced machine noise [10], noise of the propellers/propulsion [11], global resonances of the hull [12], flow noise [13] and cavitation noise [14]. The first three are the main sources of narrowband or tonal radiated noise and the latter two are the main sources of broadband radiated noise. The induced machine noise or structure-borne noise is a predominant noise source of underwater radiated noise generated by a ship at low speeds [11].

The noise radiated into the water generated by vibrations of the hull of a ship can be estimated by measuring the structural acceleration levels of the hull and applying the function that relates the measured acceleration levels and the acoustic pressure in the fluid. This function is well known as the transfer function. 

The transfer function is the relationship between the input and the output of a linear system in the frequency domain. It describes the frequency behaviour of a linear system, so that from its analytical expression, the output produced by the system due to a known input can be determined. In a simplified way, there exists two main approaches for the calculation of the transfer function for the estimation of the noise radiated by a ship: the deterministic method and the statistical method. 

The deterministic method is applied when numerical approximation of the noise radiated by structural vibrations is desired to be carried out. Numerical analysis methods such as Finite Elements Method (FEM), Boundary Element Method (BEM) or hybrid methods (FEM + BEM) are normally used. In most cases, when solving the coupled problem, FEM is used to model the equations which govern the behaviour of the acoustic wave in the fluid, whereas BEM is used to solve the elastodynamic equations related with the vibratory phenomena which take place on the ships’ hull. Due to the high computational load required by the deterministic method, this is normally used for low frequencies and small elements. 

The statistical method is mainly used for high frequencies. This approach does not require the same degree of setup details as the average noise within a given bandwidth is sought. The widely used statistical method is the so-called Statistical Energy Analysis (SEA) [14,15], which finds its maximum utility in large and complex structures. The SEA method basically works by dividing the structure into subsystems, obtaining the energy balance for each subsystem with the input power to the structure to be distributed among all the subsystems as a constraint. This input power can be obtained from an analytical method such as the mobility model. A radiation efficiency method is applied to compute the radiated sound power of each substructure, which are then combined to obtain that of the complete structure. Currently there is a wide field of investigation on vibrations and structural noise; thus, for example, in [16], a coupled FEM/BEM model is used to solve the fluid–structure interaction.

The estimation of the Transfer Function conducted by numerical modelling requires the creation of ship structure model with different levels of detail depending on the applied approach which is often very difficult because the ship geometries are not always available. On the other hand, the effort required to generate the model should be considered as well as the fact that underwater radiated noise estimations must be validated experimentally. Therefore, the need arises to use a third approach based on analytical methods that do not require such an exhaustive knowledge of the geometry of the ship and require negligible execution time, so they can be executed in real time.

This research is focused on the analysis of the underwater radiated noise generated by hull’s vibrations and its estimation by means of the transfer function using analytical methods validated experimentally. The acoustic analysis is carried out in both narrowband and broadband, covering the study of the coherence of the measurements, analysis of the machinery as an acoustic source, estimation of the transfer function and radiation efficiency and their validation by means of data collected. 

## 2. Materials and Methods

### 2.1. Data Collection

**Figure 1:**
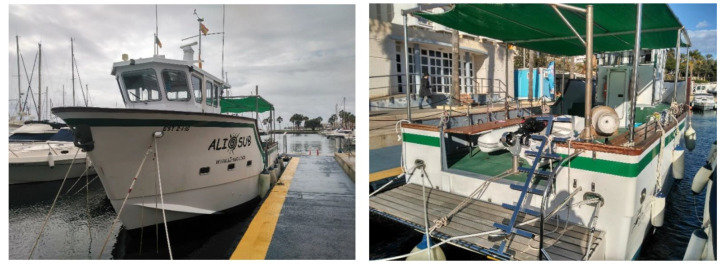
Ship under measurement with Glass Fibre Reinforced Plastic (GFRP) hull.

The measurements were collected at Villajoyosa port, close to the city of Alicante in the east coast of Spain. The ship under measurement is called Ali-Sur 5° of 12 m length. This ship is used for diver training and operations. The material of the hull of the ship is Glass Fibre Reinforced Plastic (GFRP). This material combines the high strength of thin glass fibres with the ductility and environmental resistance of an epoxy resin; the inherent damage susceptibility of the fibre surface is thereby suppressed, whereas the low stiffness and strength of the resin is enhanced. During the measurements, the ship was moored at the pier. Figure 1 shows the ship under measurement. 

To measure simultaneously the vibrations of the hull and the URN, the Vibration Control System (VCS) and Smart Digital Hydrophone (SDH) system manufactured by SAES were installed onboard the ship. 

**Figure 2:**
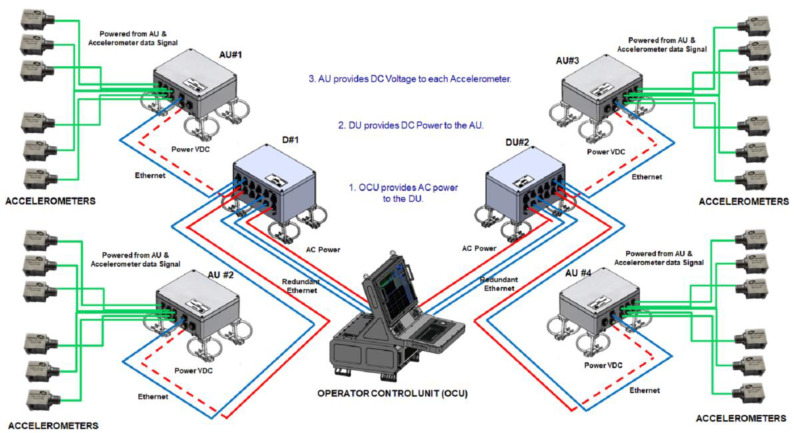
General architecture of the Vibration Control Systems (VCS) and main units. Image property of Sociedad Anónima de Electronica Submarina S.M.E. (SAES).

The VCS system is a piece of equipment based on accelerometers installed onboard the ship to measure in real time the vibrations of the machinery and ship’s hull. The two main objectives of the VCS system are to control the URN generated by the ship and to monitor the health of the structural elements and onboard systems such as motors, gears, pumps, etc. The VCS system is composed of accelerometers, Acquisition Units (AU), Distribution Units (DU) and Operator Control Unit (OCU). The accelerometers are mounted over the machinery and the hull’s panels to measure their accelerations in time domain expressed in μg. The AU is responsible for powering the accelerometers acquiring the signals and transmitting these digital signals over to the DU. The DU are the interfaces between AU and the OCU. These units are responsible for powering the AU and for data distribution over to the OCU. The OCU supports the Broadband and Narrowband Signal Processing and Human Machine Interface (HMI). The OCU is responsible for providing AC power to the DU. Figure 2 shows the general architecture of the VCS system and the main units.

**Figure 3:**
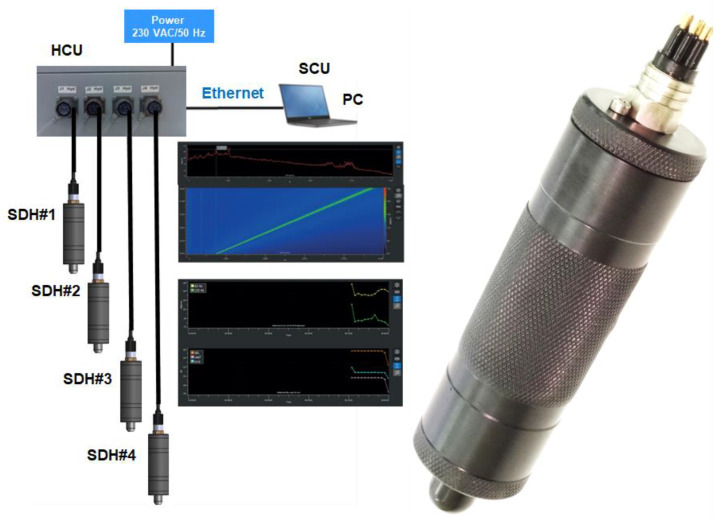
General architecture of the SDH system (**left**) and smart digital hydrophone (**right**). Image property of Sociedad Anónima de Electronica Submarina S.M.E. (SAES).

The Smart Digital Hydrophone (SDH) system is a portable underwater acoustic measurement system composed of hydrophones, Hydrophones Connection Unit (HCU) and Control and Analysis Unit (CAU). The SDH system integrates up to four (4) digital hydrophones with programmable sampling frequency of up to 150 kHz. Each digital hydrophone integrates the acoustic transducer and electronic board for acquisition and communication. The HCU allows the communication between the hydrophones and CAU providing the power supply to the hydrophones. The CAU control the data from the hydrophones, allowing their control. In addition, the CAU integrates the signal processing and analysis capability of the system. Figure 3 shows the architecture of the SDH system and a smart digital hydrophone.

The hydrophones were calibrated according to the IEC 60565 [17] and the accelerometers according to IEC 16063 [18].

**Figure 4:**
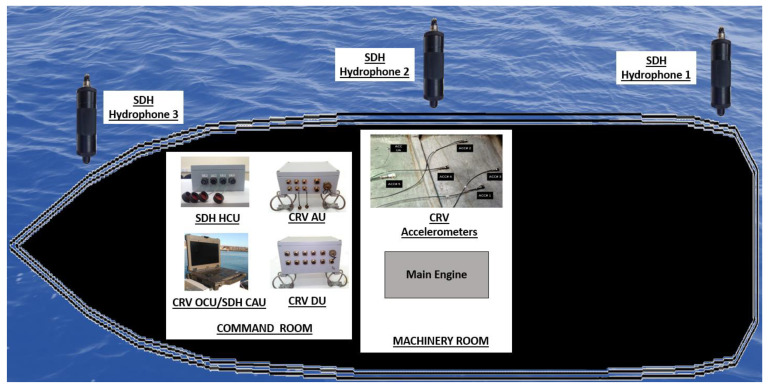
General diagram of the installed units and deployed sensors for the measurement of the vibrations and acoustical radiation of the hull and Underwater Radiated Noise (URN).

To measure the vibrations, five (5) accelerometers, one (1) Acquisition Unit and one (1) Distribution Unit of the VCS system were deployed. The acoustic measurements were performed using three (3) hydrophones and one (1) Hydrophones Connection Unit of the SDH system. To control both systems simultaneously, the Operator Control Unit and the Control and Analysis Unit of the VCS and SDH system, respectively, were installed in the same laptop. All units excepting the sensors were installed in the Command Room of the ship. The accelerometers were mounted in two panels of the hull inside the engine room and the hydrophones were deployed close to the aft, middle and bow of the ship. Figure 4 shows a general diagram of the installed units and deployed sensors for the measurements of vibrations of the hull and URN.

**Figure 5:**
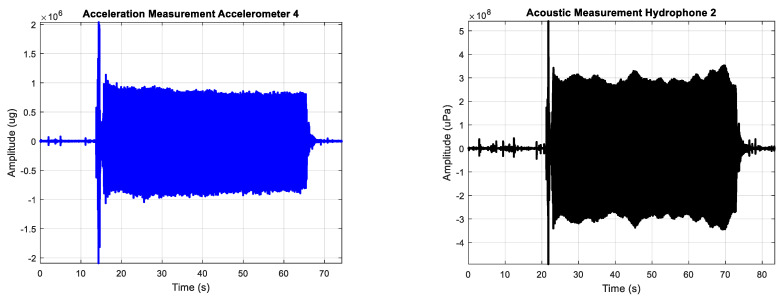
Typical time series of the measurement of the vibration of the hull in μg (**left**) and underwater radiated noise in μPa (**right**) with the main engine started.

The main engine was used as source of excitement of the hull by generating vibrations on it. Background measurements were carried out with the main engine stopped. The duration of the measurements was approximately 120 s to measure the background noise before and after turning the main engine on and off. The sampling frequency of the measurements with accelerometers and hydrophones was 32 kHz and 44 kHz, respectively. so the hydrophone measurements were resampled at 32 kHz before being processed. Typical time series of the vibrations of the hull expressed in acceleration (μg) and underwater radiated noise measurements expressed in acoustic pressure (μPa) with the main engine started are shown in the Figure 5. The starting moment of the engine is perfectly identifiable in the time series as a transient peak emerging from the background noise.

### 2.2. Broadband and Narrowband Acoustic Analysis

Underwater sounds are classified based on their duration as transient or continuous. Transient sounds are of short duration, often called pulses, and they may occur singly, irregularly, or as part of a repeating pattern. For instance, an explosion represents a single transient, whereas the periodic pulses from a ship’s sonar are patterned transients. Underwater sounds can also be classified as continuous, that is, they occur without a pause or hiatus. Continuous sounds are further classified as periodic, such as the sound from rotating machinery or pumps, or aperiodic, such as the sound of a ship breaking ice [3].

The continuous noise is composed of broadband noise and narrowband noise depending on the source. Broadband noise is a broad-spectrum noise, that is, the level of acoustic energy is a continuous function of frequency. The acoustic energy is spread to a wide range of frequencies. Typical broadband noises generated by ships are those produced by shafts and propellers, or hydrodynamic noise produced by the passage of water through the hull of a ship. Narrowband noise, as opposed to broadband noise, concentrates the acoustic energy in small frequency bands centred at different frequencies, discrete frequencies or tones. Typical narrowband noises generated by ships are those generated by machinery such as diesel engines or produced by the auxiliary equipment such as generators. The underwater radiated noise usually exists as a continuous spectrum over which are overlapped tones. Both components, continuous and discrete, decrease as frequency increases.

The reference unit for the acoustic pressure in sea water is 1 μPa, and the acoustic level N of a stationary pressure is expressed in decibels (dB) by

(1) $$N=10\log\frac{<p^{2}>}{\left(1\mu {\mathrm{Pa}}^{2}\right)}\;in\;dB\;re\;1\;\mu {\mathrm{Pa}}^{2}$$

The measure for characterising sources of underwater noise, independent of the environment in which the measurements are taken, is the Source Level (SL). This expresses the mean square sound pressure $p_{rms}^{2}$ at a distance r[m] in a certain direction in the far field of the source, where the sound pressure and particle velocity are in phase and decrease inversely proportional to the distance from the source, scaled back to the reference distance $r_{ref}=1$ from the acoustic centre of the source. The acoustic centre is the fictitious point from which the far filed sound appears to be radiated. The definition of SL can be written as

(2) SL=SPL(r)+20log(rrref) in dB re 1 μPa2m2

where $SPL\left(r\right)=10\log\left(p_{rms}^{2}\left(r\right)/p_{ref}^{2}\right)\;in\;dB\;re\;1\;\mu {\mathrm{Pa}}^{2}\;$is the mean square sound pressure level measured at distance r[m] in a certain direction. The second term of Equation (2) provides the scaling to the 1 m reference distance. The spectral density units are in $dB\;re\;1\;{\mu \mathrm{Pa}}^{2}/\mathrm{Hz}$.

The continuous noise is composed of broadband noise and narrowband noise. The broadband analysis is conducted in one third octave bands expressed as levels in a 1 Hz by dividing the one-third octave band by the bandwidth of each one third octave band. The Sound Pressure Level (SPL) expressed in one third octave band corrected by the bandwidth of each one third octave band is defined by the following equation:

(3) $$SPL\left(f_{c}\right)=10\log\left(\frac{10^{\frac{SPL_{\frac{1}{3}Octave}\left(f_{c}\right)}{10}}}{\Delta f_{c}}\right)=SPL_{\frac{1}{3}Octave}\left(f_{c}\right)-10\log\left(0.23f_{c}\right)\;in\;\mathrm{dB}\;\mathrm{re}\;1{\;\mu \mathrm{Pa}}^{2}/\mathrm{Hz}$$

where $f_{c}$ is the central frequency of the one third octave band and $\Delta f_{c}$ is the corresponding bandwidth of the one third octave band.

In this research, different acoustic analyses are conducted in broadband and narrowband depending on the objective of the analysis. The broadband analysis is performed in One Third Octave bands (OTO) according to the international standard IEC 61260 [19] and ISO 1683 [20] corrected by the bandwidth of each OTO.

### 2.3. Analysis of the Source Strength

The first criterion to decide whether the collected data are valid for the acoustic analysis or not is that the source strength must be higher than the background noise, allowing the clear distinction between these two. This analysis provides information about the capability of the engine to excite the hull panels and it is conducted through the computation of the Signal to Noise Ratio (SNR).

The SNR is well defined and understood in electrical engineering and communications [21,22,23,24] and it is frequently used in the acoustic field, especially in studies in which measurement process and instrument design are involved [25,26,27,28,29,30,31,32]. The $SNR_{dB}$ is defined as signal level minus the noise level as follows [33]:

(4) $$SNR_{dB}=SL_{dB}-NL_{dB}\;in\;\mathrm{dB}\;\mathrm{re}\;1{\;\mu \mathrm{Pa}}^{2}m^{2}$$

where $SL_{dB}$ is the Source Level generated by the engine and $NL_{dB}$ is the mean rms level of the background noise. Therefore, the condition of $SNR_{dB}$> 0 dB is met when the source level is higher than that of the background noise. 

The analysis of the source strength is performed in broadband by means of the one third octave bands processing. Because the sound levels are not scaled back to the reference distance $r_{ref}=1\;m$, Equation (4) is used with SPL levels instead of SL. 

### 2.4. Analysis of the Coherence of the Measurements

The statistical analysis of the coherence of the measurements is conducted by the study of the similarities between the vibration of the hull and the acoustical pressure in water represented by the coherence function. For two signals of pressure p(t) and vibration v(t), the coherence function is described as follows [34]:

(5) $$\gamma_{pv}^{2}\left(f\right)=\frac{{\left|G_{pv}\left(f\right)\right|}^{2}}{G_{p}\left(f\right)G_{v}\left(F\right)},$$

where $G_{p}$ and $G_{v}$ denote the corresponding spectral densities of signals p(t) and v(t), respectively, and $G_{pv}$ denotes the cross spectral density. Coherence function is a real function accepting arguments from the range of

(6) $$0\leq \gamma_{pv}^{2}\left(f\right)\leq 1.$$

Therefore, the zero value occurs for signals that do not have common source and the one value is seen for signals coming from the same source.

The analysis of the coherence function is conducted in narrowband spectrum in the frequency range from 10 Hz to 10 kHz with a 0.1 Hz resolution. Accelerometer and hydrophone signals are windowed using the Hanning window with an overlap of 50% [35], and coherence function between each accelerometer and each hydrophone for each measurement is computed. The arithmetic mean value of the coherence function is obtained across all measurements. 

The higher the coherence function (i.e., closer to value of one), the more suitable is the $SNR_{dB}$ related to that measurement; therefore, the analysis of the Coherence Function for each paired hydrophone–accelerometer measurement allows for the study of the optimal positions of the accelerometers in order to estimate the Transfer Function. 

### 2.5. Estimation of the Transfer Function

The Transfer Function (TF) relates the ship structure-borne noise and the URN and is defined as follows [11]:

(7) $$TF=L_{p}-L_{a\;}\;\;\;\;in\;\mathrm{dB}\;\mathrm{re}\;1{\;\mu \mathrm{Pa}}^{2}/1{\mu g}^{2}\;$$

where TF is the Transfer Function of structure-borne noise, $L_{p}$ is the one third octave band URN level and $L_{a\;}$is the one third octave band acceleration level. Transfer Function in linear systems is defined through amplitude and phase. In this research, the modulus square is used, not including the analysis of the phase. The radiated pressure at 1 m apart from the source under the assumptions of spherical spreading and omnidirectional directivity is defined as

(8) $$L_{p}=L_{W\;}-10\log\left(\Delta f\right)+54\;\;\;in\;\mathrm{dB}\;\mathrm{re}\;1{\;\mu \mathrm{Pa}}^{2}m^{2}/\mathrm{Hz}$$

where $L_{W\;}$ is the acoustic power level ($in\;\mathrm{dB}\;\mathrm{re}\;10^{-12}\;W$) and Δf is the bandwidth. The constant value of 54 is obtained based on the acoustic power under the assumption of spherical spreading of a point source defined as W=2πP2ρc; therefore,54=10log(ρc)−10log(2π) for water density equal to ρ = 1027 kg/m^3^ and speed of sound equal to c = 1500 m/s. Finally, the acoustic power radiated sound into water by vibrating panels is obtained by

(9) $$W=\rho_{0}c_{0}\overset{N}{\underset{i=1}{\sum }}\sigma_{rad,i}A_{i}<v^{2}>{}_{i}\;\left[W\right]$$

where $\rho_{0}$ and $c_{0}$ are the density of the seawater and sound speed, respectively, $\sigma_{rad,i}$ is the radiation efficiency of the panel i, $A_{i}$ is the area of the panel i, and $<v^{2}>{}_{i}$ is the space-time averaged velocity squared of panel i. N is the number of individual panels vibrating incoherently. 

The calculation of the Transfer Function assumes that the hull vibration radiates underwater acoustic noise as a unique source; therefore, the broadband vibration of the hull was computed integrating the OTO bands of the five accelerometers for all the measurements. The same process was performed for the measurement data of the underwater radiated noise for each hydrophone. The Transfer Function for each hydrophone position was calculated as the difference between the integrated values of the OTO bands of URN and the integrated values of the OTO bands of the acceleration. 

To evaluate the accuracy of each calculated Transfer Function, these functions were used to estimate the radiated noise by applying them to the vibration measurement data as input. The estimated acoustic radiated noise for each vibration measurement was compared with the real acoustic data. 

Before computing the Transfer Function, the validity of the measurements must be analysed for broadband estimation of the Transfer Function. The validity is analysed by means of a repeatability study based on the processing of the mean value of each third octave band of all the measurements for each sensor, and on the computation of the maximum deviation of all third octave bands for each measurement and sensor.

### 2.6. Prediction of the Radiation Efficiency

Transfer Function depends on the radiation efficiency. The Maidanik’s and Uchida’s models are used to compute theoretically the sound radiation efficiency [36]. Maidanik defined the radiation efficiency for a ribbed panel in a reverberant sound field in function of the frequency f as follows [37]:

(10) $$\sigma_{rad}\left(f\right)=\frac{4A_{rad}}{c^{2}}f^{2}\;;\;f_{11}=\frac{c^{2}}{2A_{rad}f_{c}}\left(\frac{P^{2}}{8A_{rad}}-1\right)\;\;(f<f_{11}),$$

(11) $$\sigma_{rad}\left(f\right)=\frac{\lambda_{c}^{2}}{A_{rad}}g_{1}\left(\alpha \right)+\frac{P\lambda_{c}}{A_{rad}}g_{2}\left(\alpha \right);\;\lambda_{c}=\frac{c}{f_{c}}\;;\;\alpha =\sqrt{\frac{f}{f_{c}}}\;\left(f_{11}<f<f_{c}\right),$$

(12) $$f_{c}=\frac{1}{2\pi }c^{2}{\left(\rho_{s}h\right)}^{\frac{1}{2}}{\left[\frac{Eh^{3}}{12\;\left(1-v^{2}\right)}\right]}^{-\frac{1}{2}},$$

(13) $$\sigma_{rad}\left(f\right)=\sqrt{\frac{a}{\lambda_{c}}}+\sqrt{\frac{b}{\lambda_{c}}}\;\left(f=\;f_{c}\right),$$

(14) $$\sigma_{rad}\left(f\right)={\left(1-\frac{f_{c}}{f}\right)}^{-\frac{1}{2}}\;\;\left(f>\;f_{c}\right),$$

(15) $$g_{1}\left(\alpha \right)=\frac{4}{\pi^{4}}\frac{1-2\alpha^{2}}{\sqrt{\alpha^{2}\left(1-\alpha^{2}\right)}}\;\;\left(f<\;\frac{f_{c}}{2}\right),$$

(16) $$\begin{array}{cc} g_{1}\left(\alpha \right)=0 & \;\left(f>\;\frac{f_{c}}{2}\right), \end{array}$$

(17) $$g_{2}\left(\alpha \right)=\frac{1}{4\pi^{2}}\frac{\left(1-\alpha^{2}\right)ln\left(\frac{1+\alpha }{1-\alpha }\right)+2\alpha }{{\left(1-\alpha^{2}\right)}^{\frac{3}{2}}}\;$$

where a, b and h are the width, height and thickness of the panel, respectively, P=2(a+b) is the circumference of the panel, $A_{rad}$ is the area of the panel, $\rho_{s}$ is the density of the panel, E is Young’s modulus of the panel and v is the Poisson ratio of the panel. The coincidence frequency, $f_{c}$, is the frequency at which flexural wave speed of the plate vibrating without fluid load would equal the sound speed in the fluid medium. $f_{c}$ is called the coincidence frequency and corresponds to the frequency at which the flexural wave of the plate vibrating without fluid load would be equal the sound speed in the fluid medium.

Uchida defined the radiation efficiency as follows [38]:

(18) $$\begin{array}{cc} 10\log\left(\sigma_{rad}\left(f\right)\right)=10\log\left(\frac{m\sqrt{B}}{A_{rad}}\right)-78 & \;(f\leq \;f_{1}), \end{array}$$

(19) $$f_{1}=0.25\;f_{0}\;;\;f_{0}=700{\left(\frac{m\sqrt{B}}{A_{rad}}\right)}^{0.2},$$

(20) $$\begin{array}{cc} 10\log\left(\sigma_{rad}\left(f\right)\right)=\left(\frac{50}{3}\right)\log\left(\frac{4f}{f_{0}}\right)+10\log\left(\frac{m\sqrt{B}}{A_{rad}}\right)-78 & \;(f_{1}<f\leq \;f_{2}), \end{array}$$

(21) $$f_{2}=2f_{0},$$

(22) $$\begin{array}{cc} 10\log\left(\sigma_{rad}\left(f\right)\right)=50\log\left(\frac{f}{1600}\right)-10\; & \;(f_{2}<f\leq \;f_{3}), \end{array}$$

(23) $$f_{3}=16000\;Hz,$$

(24) $$\begin{array}{cc} 10\log\left(\sigma_{rad}\left(f\right)\right)=-10 & \;(f_{3}<f) \end{array}$$

where m is the mass per unit area ($\rho_{s}h$) and B is the bending stiffness defined as

(25) $$B=\frac{Eh^{3}}{12\;\left(1-v^{2}\right)}.$$

According to the Maidanik’s and Uchida’s radiation efficiency models, the sound radiation efficiency depends on the material of the hull. In this research, the vibrations and acoustic measurements of a recreational ship with a hull made of GFRP were conducted. Both approaches are used to estimate the radiation efficiency in narrowband and broadband analysis in the bandwidth from 10 Hz to 10 kHz, and their results are compared with the radiation efficiency computed from the vibrations and acoustic measurements. 

## 3. Results and Discussion

### 3.1. Analysis of the Source Strength

The analysis of the source strength is carried out to determine if the source, in this case the engine, is capable of exciting the hull panels in the whole bandwidth in such a way that the radiated noise exceeds the ambient noise providing the frequency range of the analysis. 

This analysis is performed using Equation (4) computing the SL and the NL as the average level calculated of each OTO band for each sensor and measurement with the engine started and stopped, respectively.

**Figure 6:**
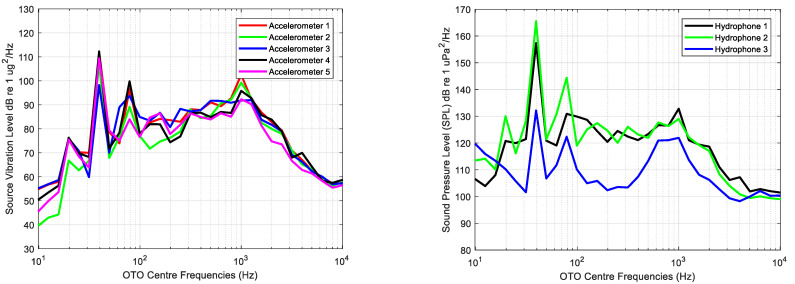
Average of each one third octave processed for all measurements with the main engine started for each accelerometer (**left**) and each hydrophone (**right**).

The average of each OTO band calculated for all measurements and each sensor with the engine starting is shown in Figure 6.

**Figure 7:**
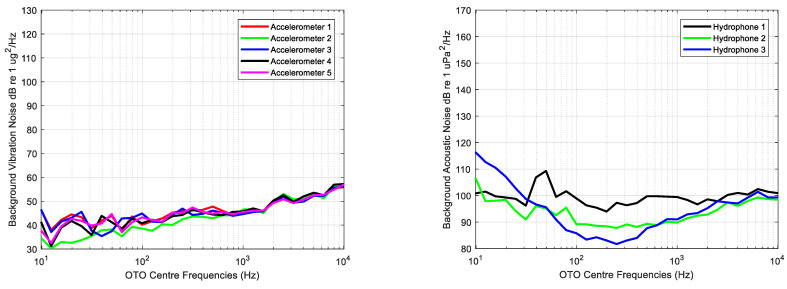
Average of each one third octave processed for all background measurements for each accelerometer (**left**) and each hydrophone (**right**).

The average of each one third octave calculated for all measurements and each sensor of background noise, that is, measurements while the main engine is stopped, is shown in Figure 7.

**Figure 8:**
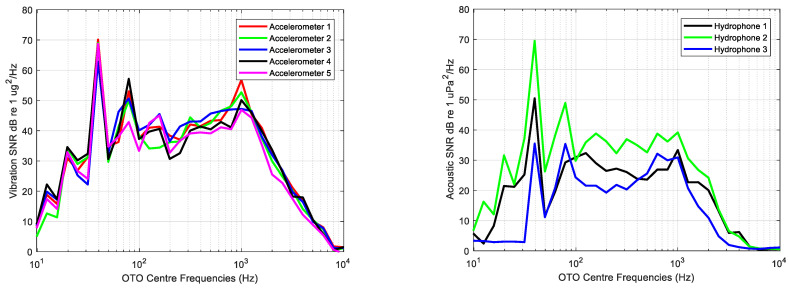
Signal to Noise Ratio ($SNR_{dB}$) computed for the average of each one third octave band of vibration (**left**) and acoustic (**right**) measurements.

Figure 8 displays graphically the ratio between the vibration and acoustic measurements of the source and background using Equation (4) without scaling back to the reference distance $r_{ref}=1$ m; the SPL levels are used instead of SL.

The lowest acoustic and vibration levels are observed in very low and high frequencies, whereas the highest levels can be observed in frequency bands with centre frequencies of 40, 80 and 1000 Hz. Variability in acceleration levels across frequency is lower than that of acoustic measurements, in which the lowest levels are provided by the hydrophone deployed at the bow.

Analysed frequencies range from 10 Hz to 10 kHz, the $SNR_{dB}$ being higher than 0 dB for all OTO bands. According to the average levels of the acoustic background noise shown in Figure 7, the hydrophone 3 levels at these bands are the highest measured during the experiment, meaning that the lower $SNR_{dB}$ is due to the existing background levels at this measurement point. 

The analysis of the source strength concludes that the levels of the measurements using the main engine as source of vibration could be useful to estimate the underwater radiated noise from hull vibrations. Specifically, according to the results of the $SNR_{dB}$ analysis in this research, the main engine can be used to excite the hull’s panels. 

### 3.2. Evaluation of the Coherence of the Measurements 

**Figure 9:**
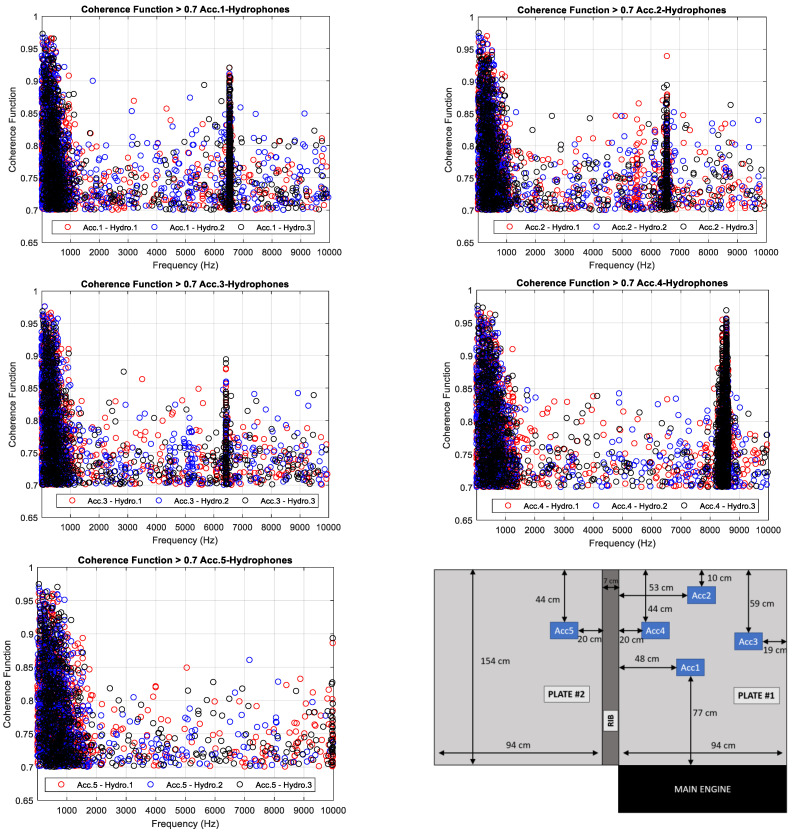
Average of the Coherence Function higher than 0.7 versus frequency between each hydrophone and the accelerometer 1 (**top-left**), accelerometer 2 (**top-right**), accelerometer 3 (**middle-left**), accelerometer 4 (**middle-right**) and accelerometer 5 (**bottom-left**). Description of the position of the accelerometers installed in the ship’s panel (**bottom-right**).

The analysis of the coherence of the measurements is carried out in narrow band and using Equation (5) with a frequency resolution of 0.1 Hz. The average of the coherence function is thresholded to obtain the frequencies for values of the coherence function greater than 0.7. Figure 9 shows the values of the average of the Coherence Function higher than 0.7 versus frequency computed between each accelerometer and hydrophone. Additionally, Figure 9 includes a scheme with the position of the accelerometers in the panels to help in the analysis of the results.

The average of the Coherence Function computed between the accelerometer 1, 2 and 3 and all hydrophones is higher than the threshold from 10 Hz to 1 kHz, increasing when the frequency decreases. This means that the source of the acoustic energy measured by the hydrophones from 10 Hz to 1 kHz is the vibration of the hull’s panels. The same behaviour is detected analysing the average of the Coherence Function computed between the accelerometers 4 and 5, but in this case, the bandwidth is expanded up to 1.3 kHz and 1.5 kHz, respectively. A broadband noise centred at 6560 Hz is clearly identifiable with a bandwidth of 40 Hz in the average of the Coherence Function computed between the accelerometer 1, 2 and 3 and the hydrophones. This broadband noise disappears in the average of the Coherence Function computed using the accelerometers 4 and 5. Two broadband noises could be identified in the Coherence Function computed between the accelerometer 4 and the hydrophones centred in the frequencies 8435 Hz and 8560 Hz with a bandwidth of 40 Hz. 

A ship is an overly complex structure, and the optimal selection of accelerometer positions to measure hull vibrations is a critical issue. It is necessary to consider different measurement points not only for accelerometers but also for hydrophones to study the vibration wave transmission through the ship structure. The Coherence Function provides the acoustic energy measured by the hydrophones and generated by the hull vibrations in the frequency domain. In this research, the effect of the rib close to the accelerometer 4 and 5 and the boundary conditions close to the accelerometer 2 and 3 are notable. The broadband noise centred in 6560 Hz measured by the hydrophones is also acquired by the accelerometers 1, 2 and 3. This broadband noise is measured by the accelerometer 4 shifted and splitted into two bands and is not measured by the accelerometer 5. These results conclude that in order to estimate the Transfer Function, spatial distribution of the accelerometers to measure hull vibrations should be focused not only on the centre of the panel, but also close to the ribs and boundaries of it. 

To study the source of acoustic energy measured by each hydrophone, the number of frequency bins for which the average of the Coherence Function is higher than the threshold is counted. Table 1 shows the number of frequency bins for which the mean of the coherence function is higher than 0.7 computed between each accelerometer and hydrophone.

According to the results shown in Table 1, the number of frequency bins measured by accelerometer 5 and hydrophones is the lowest one. The rib generated an attenuation of the vibration waves from the main engine to the rest of the ship, meaning that this position is not good to estimate the Transfer Function. On the other hand, the best position of the accelerometers to estimate the Transfer Function is that of 4, i.e., in the centre of the panel. The boundaries of the panel create similar effect to that of the rib attenuating the transmission of the vibration waves through the structure of the ship. Finally, through the analysis of the number of frequencies with a Coherence Function higher than that of the threshold computed between the accelerometer 4 and hydrophones, the attenuation effect can be observed, created by the rib reducing the transmission of the waves to the bow of the ship. 

### 3.3. Estimation of the Transfer Function 

To alternatively evaluate the accuracy of the measurements and therefore the validity of the estimations based on them, the first step of the study was to perform a repeatability analysis on the measurements. The repeatability analysis was based on the processing of the mean value of each OTO band of all the measurements for each sensor, and on the computation of the maximum deviation of all OTO bands for each measurement and sensor. Figure 6 shows the mean value of each OTO band of the vibration and URN measurements. 

Table 2 shows the maximum deviation between the mean value of the OTO values and processed OTO for all sensors in dB re to 1 μPa^2^/√Hz for URN measurements and 1 μg/√Hz for vibration measurements.

The maximum deviations of the vibration measurements (0.43–2.06 dB re to 1 μg^2^/Hz) were lower than the maximum deviations of the URN measurements (0.91–6.11 dB re to 1 μPa^2^/Hz). These results were expected since the underwater background noise was higher than the vibration noise. On the other hand, the lowest maximum deviations of the hydrophones data appeared for the measurements of the hydrophone 2 because it was deployed closer to the main engine than the others. The maximum deviations obtained were acceptable and hence the measurements were perfectly valid for the calculation of the Transfer Function.

**Figure 10:**
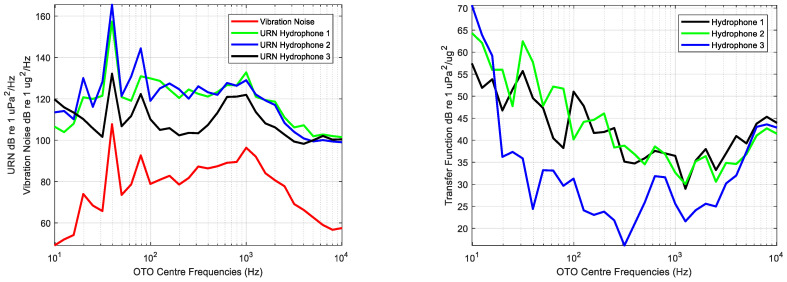
Broadband integration of the Vibration Noise in dB re 1 μg^2^/Hz and Underwater Radiated Noise in dB re 1 μPa^2^/Hz (**left**) and estimated Transfer Function dB re 1 μPa^2^/μg^2^ for each hydrophone position (**right**).

The Transfer Function calculation was conducted by processing the measurements of vibration of the hull performed with the accelerometers and the acoustic noise radiated by the hull’s panels obtained with the hydrophones in broadband. Figure 10 shows the broadband integration of the vibration and URN, and the estimated Transfer Function for each hydrophone position.

**Figure 11:**
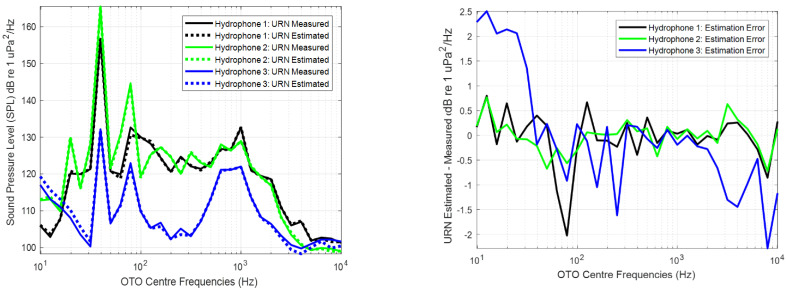
Measured and estimated Sound Pressure Level (SPL) or Underwater Radiated Noise (URN) using the Transfer Function for each hydrophone (**left**) and differences between them (**right**).

The calculated Transfer Function is used to estimate the radiated noise using as input the vibration measurements. The estimations were compared with the real measurements performed with the SDH system. As an example, Figure 11 shows the estimated and measured URN for each hydrophone and the differences between them for the eighth measurement.

The highest differences in the estimations of the URN were obtained in low and high frequencies. This is related with the bandwidth of excitation of the source. Usually, the engines do not excite the hull at low and high frequencies, so that these bands are dominated by the background noise. The underwater and vibrating noise are uncorrelated and the deviations between the estimated and measured URN are higher in the bands dominated by this noise than in the bands dominated by the source. This effect is a conclusion of the analysis of the source strength, and it can be observed in Figure 8. 

Table 3 shows the maximum and mean differences between the estimated and measured URN by each hydrophone for all the measurements.

The maximum deviation in the estimated URN is lower in the position of the hydrophone 2 because it was deployed closer to the vibrating panel. The higher difference appears in the position of the hydrophone 3 located at the bow. This is because during the measurements, the ship was moored with the bow oriented to the open waters, and therefore the uncorrelated noise in this hydrophone is higher than that of the rest of hydrophones for low frequencies. On other hand, the distance between this hydrophone and the main engine is longer than the distances between the rest of hydrophones and the engine. Finally, the effect of attenuation of the vibrating waves generated by the rib close to the panel is denoted in the difference of the estimation of the Transfer Function using the hydrophone located at the bow. To estimate the Transfer Function at the bow, the optimum choice is to install the accelerometers in the panels of the ship close to the bow.

The URN estimated by applying the Transfer Function to the real measurements of the vibrations of the hull using accelerometers has a maximum difference of 1.24 dB re to 1 μPa^2^/Hz in the position closer to the panel, 2.2 dB and 6.11 dB re to 1 μPa^2^/Hz at the stern and bow, respectively. The mean value of the difference is approximately 1 dB re to 1 μPa^2^/Hz. This denotes the accuracy of the data collected and the validity of this method to estimate the URN from the measurements of the vibration of the hull by means of the Transfer Function.

### 3.4. Estimation of the Radiation Efficiency

The radiation efficiency is computed from the data collected and both analytical methods in narrowband and broadband domain. The properties of the GFRP to estimate the radiation efficiency using Equations (10)–(25) have been extracted from [39,40,41]. Table 4 shows the values of the parameters of the seawater and panel used to estimate the radiation efficiency by means of Maidanik’s and Uchida’s analytical equations. 

**Figure 12:**
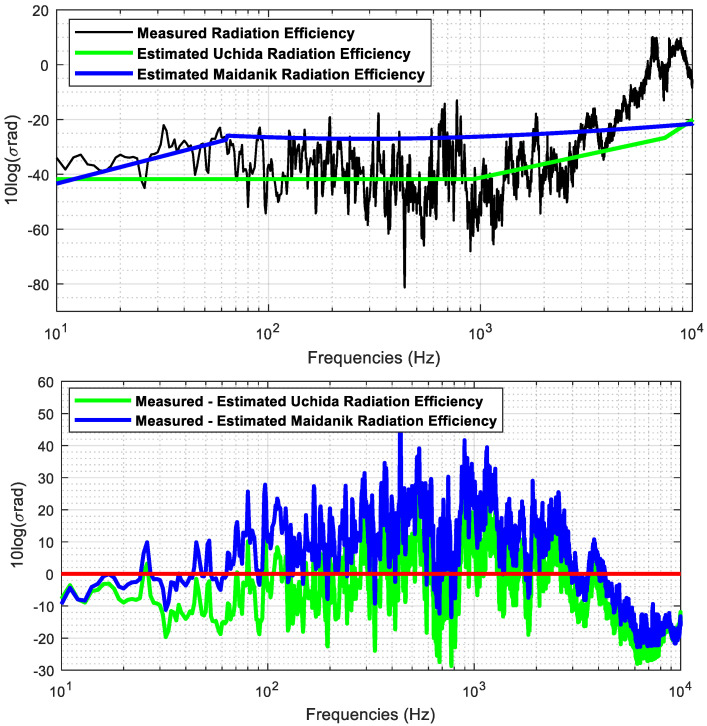
Measured and estimated radiation efficiency in narrowband domain (**top**) and difference between the estimated radiation efficiency using Uchida and Maidanik equations and the computed radiation efficiency from the measurements (**bottom**).

Figure 12 shows the radiation efficiency calculated by means of the measured data and estimated by the models of Maidanik and Uchida in narrowband domain. Additionally, it displays the difference between the computed and estimated radiation efficiency in the bandwidth from 10 Hz to 10 kHz.

In narrowband domain, the estimated radiation efficiency by means of Maidanik’s equations fits better in average up to 100 Hz. For the frequency band between 100 Hz and 3 kHz, the Uchida model is the one that provides the lowest differences relative to the radiation efficiency obtained with measured data. From 3 kHz to the corresponding final analysis frequency of 10 kHz, the Maidanik model provides the lowest differences. Maidanik’s model overestimates the radiation efficiency up to 4 kHz, presenting in average a maximum difference of 20 dB relative to the radiation efficiency computed by means of the measured data. Uchida’s model underestimates the radiation efficiency in the bandwidth from 200 Hz to 3 kHz. Both models underestimate the radiation efficiency from 4 kHz up to the maximum frequency of analysis, that is, 10 kHz. According to the $SNR_{dB}$ levels of the vibration measurements showed in Figure 8, the lowest levels are obtained from 4 kHz to 10 kHz. These low levels of $SNR_{dB}$ in the measures may appear to be underestimates in the analytic models.

**Figure 13:**
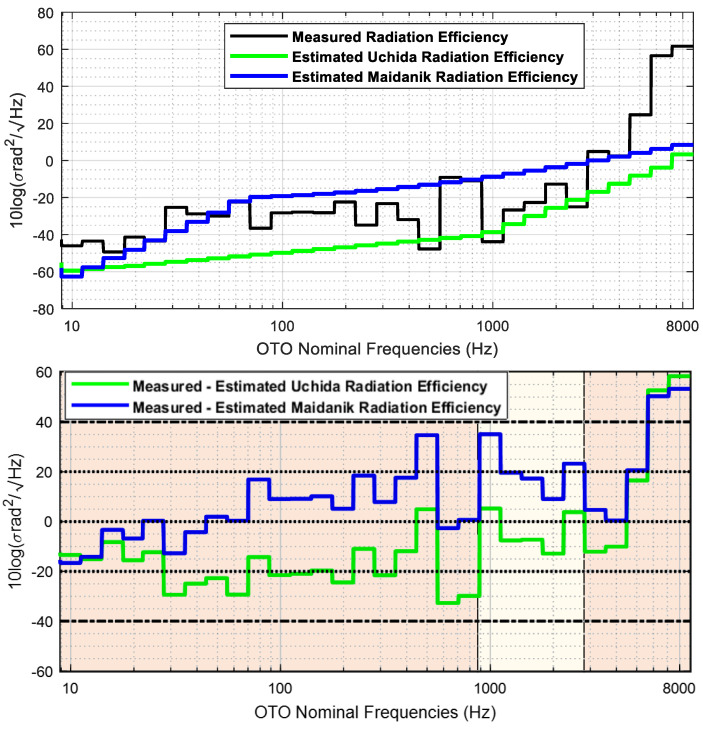
Measured and estimated radiation efficiency in broadband domain (**top**) and difference between the measured and estimated radiation efficiency using Uchida and Maidanik equations (**bottom**).

Figure 13 shows the broadband analysis of the measured and estimated radiation efficiency and the difference between them. The broadband analysis is performed computing the one third octave bands using Equation (3).

In broadband domain, Uchida’s model underestimates the radiation efficiency in the whole analysis band, whereas the Maidanik’s model overestimates the radiation efficiency from the one third band centred in 50 Hz to the band centred in 4 kHz. Both models underestimate the radiation efficiency from the OTO band centred in 4 kHz to the OTO band centred in 8 kHz. 

There are three frequency regions clearly identified in the analysis of the radiation efficiency estimation. The Maidanik equations fit better the radiation efficiency up to the one third octave centred in 800 Hz, excepting for the one third octave bands centred in 250, 400 and 500 Hz. The Uchida equations fit better the radiation efficiency from the one third octave band centred at 1 kHz to the band centred in 2.5 kHz, except for the band centred in 2 kHz. Finally, The Maidanik equations fit better the radiation efficiency from the one third octave centred in 3150 Hz up to the one third octave band centred in 8 kHz, excepting for band centred in 5 kHz.

## 4. Conclusions

The control of the acoustic radiated noise by a ship is a critical issue to mitigate the underwater acoustic pollution. The identification of the sources of radiated noise makes it possible to act on them to reduce the acoustic levels radiated into the maritime environment. 

Radiated noise can be estimated or measured at sea. The required time and resources to measure the acoustic noise at sea generated by a ship is too expensive, and alternatives solutions are adopted as the prediction of the acoustic noise by means of numerical or statistical method. Another approach is to use analytical methods which allow faster estimations compared to numerical approximations and therefore their integration into a real-time system to carry out the estimation. A ship is a complex noise source composed of the superimposition of several contributions, and this research is focused on the analysis of the acoustic radiated noise generated by vibration of the hull’s panels. 

In this research, an analytical method has been proposed that allows estimation of radiated noise generated by the vibrations of the hull by means of the transfer function using the measurement of hull vibrations. This method will allow estimating the noise radiated by a ship using accelerometers mounted in its hull. In this way, the noise radiated by a ship can be controlled in real time and reduced to mitigate underwater noise pollution. In addition, alternatively, the accelerometers mounted on the hull will allow the detection of frequencies due to breakdowns or conditions of poor maintenance of machinery and other elements installed onboard the ship.

To evaluate the method for the estimation of radiated noise, an extensive analysis of hull vibration and acoustic measurements carried out on a ship has been conducted. The analysis includes the study of the source strength, coherence function, transfer function and estimation of the radiation efficiency using measurements performed with accelerometers and hydrophones simultaneously. The objectives of the analysis are to study the ways in which the engine excites the hull panels, the ways in which the excitation frequencies are transmitted through the ship’s structure, the background noise levels with respect to the noise radiated by the panel and whether the complete measurement process has a repeatability that allows the data to be used consistently. 

According to the results of the source strength analysis, the main engine is a useful vibration source where the lowest levels are obtained in low and high frequency bands. The computation of the Coherence Function is used to determine the frequencies of Underwater Radiated Noise (URN) generated by the vibrations of the hull. Additionally, the analysis of the Coherence Function allows for the study of the transmission paths through the ship’s structure providing the best accelerometer position to estimate the Transfer Function. From this analysis, it is concluded that the best position of the accelerometers is in the middle of the panel, avoiding their mounting near the ribs and boundaries of the panel. 

The estimated URN obtained by applying the Transfer Function to the data collected on the vibrations of the hull using accelerometers has a maximum difference of 1.24 dB re to 1 μPa/√Hz and a mean value of less than 0.4 dB re to 1 μPa/√Hz. The low differences in the estimation of the URN verifies that the control of the radiated noise of a ship can be exerted by converting the real-time measurement data of the vibration of the hull to underwater acoustic noise by means of the Transfer Function. 

The Maidanik and Uchida models have been used to estimate the radiation efficiency and compare it with that calculated from the collected data. The comparison concludes that a ship is an overly complex structure and more than one method can estimate the radiation efficiency in the entire analysis band, with the combination of both models as the optimum way to analyse the behaviour of the panels in different frequency bands depending on the characteristics of the panel.

In order to control underwater noise pollution generated by maritime traffic, international standards could be developed to regulate radiated noise levels through hull vibration control. The standards should include the method of mounting the accelerometers, their location along the structure of the ship, as well as the corrective measures in case of radiating an excessive acoustic level. 

## Figures and Tables

**Table 1:** Number of frequency bins for which the average of the Coherence Function is higher than the threshold (0.7) computed between each accelerometer and hydrophone.

	Acc.1	Acc.2	Acc.3	Acc.4	Acc.5
**Hydrophone 1 (Stern)**	8290	1215	1153	5368	1191
**Hydrophone 2 (Middle)**	1234	1082	1099	1424	1059
**Hydrophone 3 (Bow)**	1119	1020	1006	1713	1029

**Table 2:** Maximum deviation between the mean value of each OTO and real values for all sensors and measurements. ID is the identification number of each measurement.

Maximum Deviation of OTO from the Mean Value								
ID	dB re to 1 μg^2^/Hz					dB re to 1 μPa^2^/Hz		
	Acc.1	Acc.2	Acc.3	Acc.4	Acc.5	Hydro 1	Hydro 2	Hydro 3
4	1.9	1.22	1.86	1.48	1.88	2.27	1.44	3.82
6	0.63	0.54	0.86	0.83	1.49	1.96	1.53	2.7
8	0.89	0.43	0.59	0.74	1.07	1.72	1	2.74
10	1.37	0.82	1.33	1.09	2.06	1.32	1.63	2.24
12	1.11	0.78	0.97	0.99	1.35	0.91	1.04	6.11

**Table 3:** Maximum and mean differences between the estimated and measured URN by each hydrophone for all the measurements. ID is the identification number of each measurement.

Differences in the Estimated URN (dB re 1 μPa^2^/Hz)						
	Hydrophone 1 (Stern)		Hydrophone 2 (Middle)		Hydrophone 3 (Bow)	
ID	Maximum	Mean	Maximum	Mean	Maximum	Mean
4	2.2	0.59	1.08	0.33	3.75	1.29
6	2	0.54	1.21	0.36	2.8	0.64
8	2.02	0.35	0.77	0.24	2.51	0.87
10	1.86	0.43	1.23	0.27	3.13	0.72
12	1.24	0.41	1.24	0.39	6.11	1.23

**Table 4:** Seawater and Panel properties used to estimate the radiation efficiency of the Glass Fibre Reinforced Plastic (GFRP) panel by means of the Maidanik’s and Uchida’s analytical models.

Symbol	Variable	Value	Units
ρ_0_	Seawater density	1027	kg/m^3^
c_0_	Speed of sound in seawater	1500	m/s
ρ_s_	Density of the panel	1900	kg/m^3^
c_s_	Speed of sound in panel	1600	m/s
E	Young’s Modulus	37.5	GPa
ν	Poisson’s Ratio	0.2	-
a	Width of the panel	1.54	m
b	Height of the panel	0.94	m
h	Thickness of the panel	0.02	m

## Data Availability

Not applicable.
